# Medicine as a Corporate Enterprise: A Welcome Step?

**DOI:** 10.4103/0973-1229.34714

**Published:** 2008

**Authors:** Murali Poduval, Jayita Poduval

**Affiliations:** * *Lecturer, Department of Orthopedics, Seth G.S. Medical College and The KEM Group of Hospitals, Mumbai, Editor, IJCP's Asian Journal of Orthopedics and Rheumatology*; * *ENT Surgeon, Mira Road, Thane, Maharashtra, India*

**Keywords:** *Business*, *Corporate Enterprise in Medicine*, *Corporate Trust Hospitals*, *Health Care*, *Large Hospital Chains*, *Managed Health Care*, *Medical Profession and Corporate Culture*, *Medicine*, *Physicians in Corporate Setup*

## Abstract

The medical profession is set for a change. It is being redesigned as a corporate enterprise. The health-care industry has proved to be lucrative and therefore has seen the entry of newer players from the corporate field into the market. The “Medical-Industrial complex” has led to the commercialization of health care well beyond what traditional practitioners would consider ideal. Medicine is being treated as a business, with cost curtailment measures and profit margins often dictating physicians' choices. A number of factors decide working environment in a corporate setup, all of which may affect the sacrosanct physician-doctor relationship and “physician” ethics. On the other side, the ability of the corporate sector to bring about a welcome change in the health-care sector in terms of availability of newer modalities of management, implementation of preventive and personalized health-care programme and, at the same time, adding to the comfort of the treating physician cannot be ignored.

When our health-care system began its strained relationship with managed care, leaders forecasted that enormous change would continue for ten years before the marketplace stabilized. Nothing could be further from the truth.

—J. H. Herndon (2003)

## Introduction

We have now entered a new era in medicine. Market forces are playing an important role in the delivery of good quality health care. As we face the onslaught of newer technology and professionalized delivery of health care, it is important to stay focused on the goal, to provide the best for our patients (Tipton, 2003).

It has been said that the health-care industry has achieved an unenviable consensus: nearly everyone is unhappy with it (Waldman, 1996). Patients complain about lack of access, insurance premiums and lack of accountability amongst the caregivers. The caregivers in turn have problems with the increasing hassles associated with delivery of quality care and heightened stress levels, while income levels tend to decline.

There is a less obvious role played by the industry that caters to the health-care segment directly and indirectly. Continuing medical and surgical education, conferences, drug and device development and research funding, preventive and personalized health care and dissemination of information to the public at large through booklets and through information campaigns – these are just some of the domains in which the corporate industry can have a role to play. Personalized health care is a domain that has been developed in the era of the corporate hospital, assisting the physician to add to the patients' level of comfort while delivering the best of treatments.

With the era of the corporate hospital, there came the advent of five-minute medicine and the decline of patient care (Tipton 2003).

This is but one point of view. One has to consider the other side of the story too. Corporate medicine allows a number of specialties and super specialties to come under one umbrella to provide consolidated and integrated care to the consumer.

It also allows the patient to utilize his medical insurance packages and group health schemes optimally with the least amount of hassle. Many companies today have group insurance packages for their employees with a large number of hospital chains enrolled in their networks. The insurance companies, too, offer similar insurance schemes. Professional bodies like the Association of Medical Consultants have also started group insurance and cash-free insurance packages for their members through third-party administrators. These schemes indeed add levels of comfort that were so far not available to the patient or physician. Corporate medicine has a role to play in continuing physician education, continuing surgical education and, most of all, in disseminating information to the patients appropriately. Last but not least, it is impossible to ignore the role of industry in development and trial of new medicines, devices and medications.

The role of corporate medicine in clinical research is here to stay. Medicine, therefore, is increasingly a corporate enterprise. It is likely to benefit the caregivers and their consumers with a positive impact, the early reverberations of which are being felt in our country, India, today. Some fields of corporate involvement in medicine are listed in Table 1.

**Table 1:** Corporate involvement in medicine

1.	Managed health care
2.	Medical tourism
3.	Diagnostics
4.	Medical education
5.	Clinical research and development
6.	Personalized health care
7.	Health insurance
8.	Drug development
9.	Patient education

## Managed Health Care

Health care as it is practiced now has its roots in the early period of the last century, where the American Medical Association paved the way for the establishment of the Blue Cross and the Blue Shield to pay for hospital costs and physician costs respectively (Mamdani, 2001). Thereafter, the development of Medicare in the 60s and subsequently the establishment of HMOs (Health Management Organizations) set the stage for the professional management of health care. The aim of reducing health-care costs and improving productivity meant that HMOs could check the rising costs by reducing inappropriate utilization of hospital resources and physicians while improving preventive measures such as vaccinations, screening for breast and colon cancer, etc. (Mamdani, 2001). The principle of universal health care allowing the insured to check his coverage plan was proposed much later.

Managed care combines financing and delivery of health care in a single entity. The managed-care organization holds contracts with physicians and hospitals allowing its clients to access these facilities through a “gatekeeper,” usually a nurse, who decides if such access is needed or not. The physicians in turn need to follow specific guidelines as well as regulations on use of diagnostic and prescription patterns as dictated by the HMO (Mamdani, 2001). The Gatekeeper system has come under flak for restricting physician access, therefore setting the stage for potential complications.

A number of problems are known with these setups. Geyman (2003) examined the effects of corporatization on costs and access to care. He noted that the corporatization of American medicine has extended into all aspects of the health-care system. The onset of hospital chains as investor-owned, for-profit hospitals is an offshoot of the corporate transformation of medicine. They may be a small part of the heath delivery network, but their influence is considerable. They have a tendency to occupy markets that produce profit rather than concentrate on community needs. The costing of these hospitals is 3-11% more, spending more on administration and ancillary services (Geyman, 2003). Despite their inflated bills, their services are often poorer. Leverage of for-profit hospitals is enhanced by a) ownership and management of laboratories, rehabilitation and long-term care services; b) asserting clout in rate setting against insurers and HMOs; c) setting up own health insurance plans; d) lobbying at the government level to further own interest; e) also, these hospitals are less likely to provide care for the non-insured (Geyman, 2003).

The reality is that with managed care we have the seemingly paradoxical purposes of saving money for the government while making profits for the managed-care corporations – indeed, a mutually beneficial transaction for the powerful parties involved (Faria, 1998a). The doctors, the ones giving the care, are used by both; while the patients, sick and vulnerable, are left out of the equation (Faria, 1998a).

Dr. Faria takes the argument further saying that HMO policies impose a gag order on the physician to reinforce the decision of rationing of care and limiting their ability to use diagnostics as they see fit. Limited communication with patients often makes patient care very impersonal and noncommittal, leaving the patient dissatisfied. This problem of having the consultant beholden to the hospital for ‘allowing’ him to have a practice at all is a disturbing trend. There have been tales told of consultants being shown graphs of their monthly performances with details of utilization of facilities and income being discussed in graphic detail. All these lead to a blatant conversion of a ‘human profession’ into a ‘commodity’; the very thing we were trained to avoid. The human touch was found to be missing (IJME, 2001). Corporate rivalry often puts tabs on doctors joining other institutions or having another practice in the vicinity, thus hampering his ability to broaden his practice (IJME, 2001). Board of directors and health-care managers tend to view physicians as uncooperative, government regulations as excessive and costs as uncontrollable (Waldman, 1996).

The health-care system is direly in need of change. Systematic health-care reforms and prudently applied corporate principles hold promise for renewing public trust in the ability of health-care providers and organizations to provide quality health care at a reasonable cost (Waldman, 1996). This has become necessary in view of recent media reports on accountability of certain corporate health-care institutions for recovering costs. There was considerable argument as to how justified was the hospital in recovering the costs accrued in treatment if it did not succeed. Therein is raised the question, “*Can we view medicine as a business?*”

The committee for costs of medical care (CCMC) called the hospital as a place of business, its business being medical care. The hospital, however, is a little different. It needs to be a hotel, an industrial plant, a repair and rehabilitation center, a haven of refuge and often, an educational institution (Perkins, 1998).

### Is Medicine a Business?

What has allowed this egregious corporate penetration to take place in an ancient, beneficent and revered profession that had held itself together as a sacred calling for centuries? (Faria, 1998a)

Entering the medical profession is a sacred calling for all of us in the community of physicians. Somewhere along the way to achieving a practice based on compassion and ethical evidence-based medical science, comes the realization that it is also the means to make a living. This becomes a rather difficult situation, wherein one has to be humane and, at the same time, deliver the highest standards of care and make money too. This equation is never as challenging as in the field of medicine – hence the difficulty in viewing doctors as one would view another professional. Isn't the doctor just another guy trying to do his job? Isn't this his “business” too? Just as it is erroneous to look upon doctors as noble saviors or, worse still, gods, it would be simplistic to hurl allegations against the medical community for being greedy businessmen. Human values, in general, are undergoing steady erosion. Consumerism and materialism, getting ahead in life and being the envy of friends and colleagues, are the rules by which most of us live today. The corporatization of medicine is the natural outcome of the way society is evolving. First, the culture of business and profit making is entering the medical establishment at all levels, starting right from medical education itself. The complete collapse of quality medical education in the form of substandard, poorly equipped medical colleges is perhaps causing the maximum harm. Students pay huge capitation fees and then go on to try and recover their expenditure by unscrupulous means. Some end up as quacks, some attempt to eke out a living through fair means but succumb to filthy lucre, some buy their way into a corporate setup to gain anonymity, while some completely dissociate themselves from clinical practice and seek other means of livelihood. So that leaves out a pathetically small number of medical professionals who, though not noble in every sense of the word, nevertheless do their fraternity proud. Even two decades ago, there were only government-run medical colleges with teaching staff that was considered one of the best in the world, not only in terms of knowledge but also in imparting to students the correct clinical approach to patient care. This approach to patient care is slowly but surely fading from the training of medical graduates today. Even if a course on ethics is introduced into the curriculum, it may not be able to substitute for the culture of being ethical that was ingrained into students by their teachers and seniors through five and a half years of medical school.

If being noble is what one should strive for, then why not become teachers and social workers? And why is it so important to be noble? Why should one not study or work for the genuine love of the subject and not because society thinks it is lofty? Why can one not perform one's work with passion instead of being burdened with the dubious distinction of being in a noble profession? Let us ask ourselves if the tag of nobility is relevant at all to the profession. A doctor deals with a patient's health, a lawyer with his rights, an engineer with his comforts, an architect with his living space and so on and so forth. Each one must do his part by using his knowledge for the furtherance of mankind, and each worker must enjoy dignity of labour—from the menial worker to the professional. This hypocritical perception of the medical profession as gods and heroes has allowed the unregulated growth of medical colleges, a good number of which lack clinical facilities. What then can we expect of the scores of graduates every year? They have little or no real skill to treat a sick person, but nevertheless have to make a living. There is a doctor in almost every building today, and all have to survive and grow. While they use their questionable resources to try and heal, it is anybody's luck that the patient gets better.

Second, the people involved in medicine-related occupations – namely, the pharmaceutical and biomedical industries – are preoccupied mainly, if not only, with profiteering. Doctors have to compete and the pharmaceutical companies have to thrive. To do this, pharmaceutical companies will go to any extent to please and doctors will never have enough! The money spent on treats for the doctor gets translated into increased cost of medicines. To keep prices in check, corners are cut, expenditure on drug research is reduced, existing drugs are modified and manipulated to be touted as new molecules, adverse drug reactions are kept under wraps and marketing gimmicks adopted to popularize the drug, until one fine day a disaster that was just waiting to happen actually occurs; and the credibility of the drug, the doctor and the pharmaceutical company crumbles like a pack of cards. Who gains? Well, of course, the litigants and the patient, although his victory is linked to his misery. Who gets away? The government and the policy makers – they still go back to passing the buck to the doctors and drug makers for not being ethical, forgetting all the time that wrong policy decisions and a lackadaisical attitude were also part of the bigger picture.

Third, the information available to the layman now comes from not only his doctor but also his acquaintances, the internet and the media; with the result that he feels empowered, but not quite. Most patients are eager to know more about their problem from their own doctor, but a good number would not hesitate to challenge the doctor on his line of treatment and also not only seek a second opinion but try alternative methods of medicine as well. When everything fails or a calamity occurs, it is usually the first doctor who faces the brunt of the patient's ire, both by way of litigation and refusal of fees. The consequence is that the doctor in the small nursing home or independent clinic feels increasingly threatened and insecure.

Indemnity cover and a corporate setup afford security in numbers, and more and more doctors are attracted towards the relative safety of a big hospital – complete with armed security personnel round the clock, public relations officers to handle the messy cases and finally, the corporate top brass who engage the services of the best lawyers to exonerate them.

All this obviously involves big money, and who else to bear the cost other than the consumer! The positive aspects of a corporate hospital, such as access to different specialties and amenities under one roof, five-star comfort and availability of the latest equipment and technology, often get overlooked in the face of prohibitive costs. Thus only the rich and/or the insured can afford these hospitals, while the rest can only whine; whereas the media is always on the lookout for any untoward incident connected to the hospital. It is no wonder then that almost everyday one comes across juicy gossip and sensationalized news about some or the other form of malpractice. Innovative treatments, fascinating surgeries, outstanding doctors are much less visible – either in print or television.

## Medical Profession and Corporate Culture

Medical profession and corporate culture – are the two concepts mutually exclusive? From the era of the humble family physician who was the end point of all of a patient's needs, we have progressed to the era of the five-star corporate hospitals with hi-tech facilities but inadequate patient satisfaction. Let us not forget that medical science is about healing people. People involved directly in this enterprise are doctors and paramedical staff; and those indirectly are the pharmaceutical industry, services staff, medical equipment industry and medical institutions. Each spoke in the wheel of health care has its designated function, is indispensable and has an equivalent role to play in optimal health-care delivery.

At the center of the wheel is the doctor. A humane approach by the treating physician not only ensures proper treatment but also elevates the doctor to the status of a demigod. Why is it that there are big tertiary care hospitals with such a reputation that patients flock to them from far and wide while some others are simply five-star hospitals catering to the rich and the insured?

The culture present in successful business enterprises therefore needs to be implemented to make medicine a successful business. If health care were deemed a business, then the tools that help fix other businesses will fix it too (Waldman, 1996).

Can big corporate hospitals do this? Why not? It would be all too easy if everyone involved at every level remembers that the hospital exists because patients have to be treated. Starting from the receptionist at the entrance to the administrative staff to finally the medical and paramedical personnel, everyone should treat the patient as a revered guest. Pharmaceutical companies, medical equipment manufacturers, philanthropic bodies, insurance companies and finally the government should work in tandem with doctors to ensure quality care and to subsidize poor patients.

A corporate hospital may have all the trappings of a five-star luxury hotel, but a good number of such hospitals are found to be abysmal in terms of competency and adequacy of treatment. This is reflected in the increasing incidence of litigation against doctors and hospitals. Let us not forget that the patient is not a fool and is not to be taken for granted. A patient may be brought to the hospital on his deathbed and may not be saved despite the doctor's best efforts; but if there has been sufficient communication and a humanitarian approach shown to the family, very few disputes would arise as far as settling the bill is concerned. Not only doctors but also paramedical and administrative staff should be trained to handle such situations.

### Low Payoffs to Physicians and Patient – The Final Recipient

Achievement of value and profitability through emphasis on efficiency, productivity and high quality is not necessarily seen as a feature of health-care delivery setups (Faria, 1998a). Despite inflated costs, the for-profit hospitals are often shown to provide inferior quality of care (Geyman, 2003). The term “medical-industrial complex” to describe these interrelationships is not a new concept, the term having been introduced in 1980 (Relman, 1998; IJME, 2001). It implies a new industry that supplies health services for profit (Relman, 1980).

The evolution of the corporate hospital came from increasingly low payoffs to physicians from Medicare (Relman, 1980; Kereiakes, 2004). Physicians sought comfort and safety in numbers by resorting to group practice in the face of increasing litigation and practice costs. Although the corporate hospital gave a space for practice, it increasingly encroached on the physician's autonomy and forced him to compromise to curtail costs (Kereiakes, 2004; Faria, 1998b). This tendency to compromise is what ails the health-care system (Waldman, 1996). Even if health care is more a public service than a business, management principles and solutions can apply. A bridge must be built between the two cultures.

It is often assumed that “hospitals function like other businesses, meaning high costs equal inefficiency” (Relman, 1980). This needn't apply to the health-care or hospital setting. A common example often cited is that of joint replacements. The cost of joint replacement is escalating with the cost of implants; however, the payments from care providers have remained almost static. This means that the bulk of the package goes towards implant costs, decreasing profits and physician payouts. To offset this, if a cheaper implant were to be used, the longevity and safety of the joint would be compromised, which is hardly the correct solution to curtail costs. This type of cost curtailing measure is often used to provide joint replacement solutions to uninsured and poor sections of society, in public health setups and in some private hospitals. Short-term gains are always attractive; but what does one say of long-term losses, of the higher incidence of revisions to be expected out of such irrational use of surgical procedures? A more rational solution is to include joint replacement in the ambit of general insurance more widely and also to ensure good local implant quality by implementing certification and continuous-monitoring procedures. The payouts in packages by the insurers must make provision for increasing costs.

As a means to achieve efficiency, corporate strategies of TQM (total quality management) and CQI (continuous quality improvement) may be applied in the health-care setting. However, these strategies have not found equivalent success (Waldman, 1996).

Every aspect of health-care delivery and ancillary services is inextricably linked to its final recipient, the patient. This equation changes the very perspective with which we view the so-called “enterprise.” Our clients are human beings in physical and mental distress, and disease takes a heavy toll on emotions and well-being of everyone in the vicinity of the sick person. Profit-making, therefore, has to be weighed well against the comfort and care provided to the patient and his near and dear ones. Under no given circumstance can patients' concerns be allowed to be pushed aside in favour of curtailing costs and achieving profits.

### Corporate Trust Hospitals

There have been accusations of corporate hospitals run by trusts not fulfilling their obligations of providing free treatment to a certain percentage of patients as specified by law, while claiming all exemptions that can be claimed on tax and equipment. It needs to be noted that none of these so-called trust and research centers do any research. It would be enlightening to know their quantum of research output, besides that measured in rupees, and their research setups before permitting further sanctions.

None of these trust hospitals are cheap either. Most of them are as expensive as the hospital next door. The only doctor who treats “cheap” in the private sector is still the nearby “small nursing home.” The only thing in which the charitable and trust tag comes up is in the physician payout, which is miserable, to say the least. It is also quite educating to see the small proportion of the total package that constitutes the doctor's charges. Charity is practiced by the doctor, not the hospital, and the hospital practices charity in the name of the doctor.

### Corporate Culture in Health Care

Health-care organizations are social groups comprised of people who pursue a common purpose, share values and beliefs and therefore possess a common culture (Waldman, 1996).

Corporate culture is fundamental for accomplishing any sustainable change in care delivery. The term implies that the altruistic call of healing has a business side too (Waldman 1996); and by culture, we mean values, attitudes and behavioral attributes.

Waldman (1996) feels that there is a threefold reason for the problems in corporate health care:

1. If the corporate culture in health care is seriously dysfunctional, it could be the root cause of its problems.
2. The human resource development issues in health care are of concern because of high turnover rates and professional withdrawal.
3. Corporate culture tends to resist change. In medicine change is homeostatic and essential for functioning and doesn't always translate into costs.

Sometimes, management principles such as optimization, which otherwise works well in other cultures, may have exactly an opposite effect in health care. The apparent cost cutting by cutbacks in staff and diagnostics, as per corporate management strategy, may actually lead to delays and increased costs as compared to savings which were expected.

### Physicians in the Corporate Setup: Redefining Roles

There is a significant dissent about the role of the physician in the corporate setup. Relman defined the medical-industrial complex so as to include the complete range of businesses concerned with the delivery of health care (Relman, 1997b).

The managed-care profession is only a part of this industrial complex. Insurance is a major subsidiary, with the premiums paid for health care forming a major chunk of health-care expenditure. A large number of cases are paid for through fixed case payments, the physician being paid on a fee schedule decided by the hospital. As a result, there is a tendency on the part of the hospital and third-party provider to restrict the use of expensive investigations and to restrict the access of the physician to the patient. This is a move to curb costs and maximize profits on a case-to-case basis. Relman emphasizes on the very important relation between the patient and the doctor and says that there are definite signs of dissatisfaction amongst both parties (Relman, 1997b). The current discontent amongst physicians in the market-oriented system is very evident. The reason is the increasing corporate control over their profession. He predicts that physicians would release themselves from the stranglehold of the corporate industry and reassert the sanctity of the doctor-patient relation (Relman, 1997b). In an attempt to get doctors to play an active role in the administrative procedure, gain-sharing procedures were adopted at some places (Healy, 2006). This implies that the physicians have an incentive for decreasing hospital costs and generating profits. Whereas this may imply a fallback on ethical practice and tempt the physician to resort to cost-cutting measures, it has been proposed that quality control and disclosure of interests would prevent problems in gain-sharing practices.

Another related issue is the role of the physician-entrepreneur in a corporate hospital setup. This year (2007), in the month of May, a senior cardiac surgeon was ousted from the very hospital he had built and sustained over the last 20 years. This hospital had changed hands recently and it found that its plans for a Medicity clashed with the good doctor's own plans of setting up a Medicity in the same region. He was summarily sacked without as much as a decent warning when talks proposing a merger between the two projects failed, causing considerable bitterness between the two parties. There was a week of high drama, with both parties trading charges; the court intervened and granted the surgeon a reprieve of a couple of months to continue his work. Shortly after this he was reinstated, the whole thing having been put to a misunderstanding. Even more surprising, this surgeon sold off his 10% stake in this hospital to the same people who had sacked him and went and joined a rival chain of hospitals.

There are a number of issues here, the most important being the importance of the doctor in the corporate chain of hospitals. It does seem from such incidents that the doctor is just another spoke in the wheel, not the most important gear in the hospital machinery. The age of “Doctor as God” seems to be at an end. “*The new breed of Indian hospitals seems to be marrying trends with facilities, offering patients the best of both. Where then is the place of the ‘Devta Doctor’ in these new ‘Mandirs of Medicine”?*” (Sengupta and Dasgupta, 2007).

Ms. Suneeta Reddy of the Apollo group was quoted as saying, “When medicine was an art, doctors were gods. But now it is more and more a science and that needs all manner of clinical inputs. That is why hospitals have become the critical factor” (Sengupta and Dasgupta, 2007). The same article emphasizes that the doctor cannot be bigger than the brand. The marriage between the *doctor-brand and the corporate-brand* has to be perfect for it to succeed.

Differences comes up when the doctor is no longer treated as god but just as another service provider; that's when he loses brand value and the sustainability factor comes into play. The sustainability of a corporate hospital has to be independent of the brand “doctor” for it to be viable, and the doctor has to be prepared to accept this fact for accessing the facilities and conveniences provided there.

### Large Hospital Chains – Apollo, Max, Fortis, Wockhardt

There has been a huge interest in the health-care segment in India in recent times. Large hospital chains have spread all over the country. The basic health-care setup was the small nursing home or the trust hospital in the past. This has now given way to a number of hospital chains like the Apollo, Max, Fortis and Wockhardt hospitals. These have not only penetrated big cities but they also cater to smaller towns and districts by way of satellite clinics or smaller outreach programs.

Take the example of Apollo, which holds about 19 hospitals and has a foot in the pharmacy business too. It caters to primary-, secondary- and tertiary-care units, with the primary- and secondary-care units acting as feeders for the tertiary- and specialty-care centers. Bed strength is in the range of about 3,500 beds. It is the leader in tertiary care, and its Chennai and Kolkata facilities have a significant market share. It is now establishing a global presence – with units in Bangladesh and Colombo, staffed by a significant number of Indian consultants. There is a preference, noted by market analysts, to focus on tertiary care (as it is more paying) and to appoint doctors as full-time employees, as it is a major determinant in limiting costs and increasing turnovers. Historically, a revenue-sharing agreement was the norm. It has been shown by market analysts that as much as 28% of costs are employment costs.

The Fortis group has established 10 hospitals and 12 heart centers in 5 years, bed strength of close to 1,600 and a stake in the Escorts Heart Institute. Wockhardt has also entered the hospital business with as many as 5 hospitals. Max is a subsidiary of the Max Group and has 4 functioning hospitals.

It is to be noted that all these are run by major players in the pharmaceutical and health-care segment. For the doctor, this has created a never-before opportunity to practice in plush environments and offer world-class care. He has access to the best equipment, excellent trained medical staff and public relation and marketing support, without having to bother about any personal investment. Costs are still prohibitive and yet are offset by an increasing number of employers opting for these hospitals as preferred health-care destinations for their employees and paying for costs through group insurance policies. This not only makes health care more accessible but also generates a regular income for the hospital. Cashless insurance policies are also a major determinant in choosing these hospitals for care.

The focus has therefore shifted away from the small nursing home. For the patient who has no access to insurance and who has no employer paying for him, the choice is still difficult and expensive; and probably the small nursing home may provide answers for his basic health needs. For the rest, the bigger corporate hospitals offer an attractive advantage.

## Medical Education and Disseminating Information

The corporate industry has been very instrumental and supportive in conducting Continuing Medical Education programmes and in updating physician skills. Continuing medical education and surgical workshops are essential today for remaining up to date on the current trends in medicine and surgery. It also keeps you abreast of what's happening in the field, as well as gains you accreditation points.

Conducting CMEs and workshops is not easy for any professional body or individual. Right from faculty to delegates, everyone expects to be pampered in the name of education and not pay for it. It is at this stage that the industry walks in with its vested interests veiled in the cloak of academic sincerity. We cannot do without corporate sponsorships and it does help us run our conferences and CMEs well, so why not accept it as inevitable? At the same time, it is essential that organizations and members remember to keep their conflicts of interests clear and transparent, without succumbing to commercial exploitation in the name of education. Dr. Magotra says that there is a fourfold reason for the current state of affairs:

First, doctors belong to a noble profession and their professional organizations have lofty goals and objectives.

Second, attending conferences and updates is absolutely essential for doctors to keep up with the desired professional standards.

Third, modern medicine is controlled by high-tech sophistication, which is expensive, and therefore generates fierce competition between companies and consequently, generates marketing pulls and pressures.

Fourth, doctors become unwitting collaborators of these companies and are at a significant risk of getting influenced while making treatment choices for their patients.

Finally, the increased costs are directly passed on to patients (Magotra, 1997).

Erosion of values has seeped into the medical system as much as in other fields, and it has been oft repeated in the press that doctors are accepting “unethical” sponsorships. Are doctors, as members of a so-called “*noble profession*,” immune to needs and requirements of daily life? Can they not, as responsible members of society, claim the benefits of a comfortable corporate sponsorship for their medical conferencing and educational needs, without having any vested interests and after declaring a clear conflict of interest where necessary? Why not?

Knowing the paucity of funds for educational activities at government hospitals, they should actively encourage corporate involvement in these institutions. There can be alliances for support of research and projects and these can translate into funds to present these findings in conferences and meetings. This would allow better dispersal of funds to more candidates (Magotra, 1997).

Academic medical alliances include the development of an internal administrative process to develop, manage and implement corporate alliances; design the optimal structure for these alliances and proactively negotiate the partnership agreements (Melese, 2006). The oversight of such a mutually beneficial exercise in academic medical centers would be in sponsored research agreements, sponsored clinical trials, material transfer, technology transfer and upgradation, continuing medical education and continuing surgical education. Such alliance practices need to be managed with individuals who have extensive experience in health care, pharmaceutical and managed care industries (Melese, 2006). These managed partnerships could be an answer for many of our ailing tertiary institutions and secondary care hospitals. Organizations exist that develop and promote ‘best practices’ for alliance practitioners in the corporate sector and such practices will be highly beneficial if adopted in academic medical centers (Melese, 2006).

## Solutions and Arguments

An earlier issue of the *Mens Sana Monographs* tried to bring things into perspective with respect to the relation between patients and doctors in the corporate structure of medicine (Singh and Singh, 2005-2006).

If medicine were to be treated as a *corporate enterprise*, then medicine would turn into a business, with patients being its clientele. Research might be guided by profits rather than by real needs. The importance of making profits may go hand in hand with the ability and capacity to devote some funds for socially relevant causes. The ethics followed are those of business and need not always be those of a profession. The biggest challenge here would be to achieve a right balance between profits and patient welfare without inherent conflicts. It is likely that better and more efficient medical services would develop from this arrangement, with an ethically and professionally satisfying relation between the patient and his doctor. However, the availability of ideal services is likely to be associated with some hitches, which include inevitable cost escalation; this may be controlled by intense competition between several such health-care enterprises. The second hitch is neglect of socially backward classes and their ailments. Now, this particular problem has to be solved by a policy of self-discipline and self-regulation, with appropriate governmental legislation, to consciously avoid neglecting any specific economic group or ailments specific to any population. Thirdly, it may often be difficult to regulate certain unscrupulous people taking advantage of such a system to reap unhealthy profits. Lastly, it is difficult to control market forces that often regulate business decisions and management practices.

On the other side is medicine as a *patient welfare centered profession*. This is a utopian situation, where the patient is foremost and profits accrued are considered a bonus, if and when achieved, and an attractive perk – not the only objective of the practice.

The ideal would be somewhere in between – a more practical solution of converting medicine into, what the authors have called, a *Patient Welfare–Centered Professional Enterprise*.

Here patient welfare must assuredly take pride of place, and one has to assure quality of services. Profit needs to be also made with these primary criteria firmly in place. In other words, “We think a healthy proportion is patient welfare *with* profits, in that order – such amount of patient welfare as also ensures profit and such amount of profit as also ensures patient welfare.” Because: ‘*Profit without patient welfare is blind. Patient welfare without profit is lame*’ (Singh and Singh, 2005-2006).

## Conclusion

Corporate involvement in medicine is here to stay. We need to work it into the system than try working around it. There is an obvious conflict of interest between the physician-patient relationship and the corporate tendency to cut costs at the expense of optimum patient care. It needs to be understood that medicine, as a humane profession, does not lend itself to management practices in the same way as other industries. The patient is paramount and the goal should be common – achieving optimal patient care, easily accessible medical services, single-window assistance and minimizing error rates. One must ensure such amount of patient welfare as also ensures profits and such amount of profits as also ensures patient welfare.

As we began with a quote from Dr. Herndon's address to the American Academy of Orthopedic Surgeons, I find it appropriate to quote him once again. He says, “*It is a known fact that we (Americans) practice good surgery, but it is necessary to do better*” (Herndon, 2003).

What we risk by doing less or inappropriate treatment is the trust and faith that our patients have in us. As Herndon says further, *“one more turn of the wrench. And, I think that is exactly what is needed in medicine. One more turn of the wrench – by each of us, by all of us. One more turn of the wrench to ensure quality.”*

At the same time, it is essential not to lose sight of the fact that for all the effort put in, the fees paid to physicians must be compatible with the charges laid by the health-care provider. If the physician has a stake in the hospital, he must ensure he adheres to ethical practices in the course of conducting his duties as a doctor. Corporate alliances have definitely improved the levels of care available to the patients at large; but worldwide, concerns have been raised about the conduct of these organizations with respect to patient care. A lot has been said about the limitation of physician access and the violation of the dignity of the doctor-patient relationship. These put together still do not disprove the fact that managed health care and corporate involvement in medicine is here to stay. We as physicians need to ensure that we are able to conduct ourselves in a manner worthy of our profession, within these institutions, without succumbing to commercial manipulation of the quality of patient care but at the same time securing adequate compensation for our services. This would, in effect, lead to more doctors entering the administrative aspects of managed care in health in the future. As for the present, corporate involvement in health care is set to be a prominent and influential fixture on the health-care horizon.

### Take Home Message

Medicine as a profession will always be linked to its prime beneficiary, the patient. This basic feature distinguishes the health-care industry from other service-based industries. Here, one cannot de-link patient welfare from other features like profit and cost curtailment. This can often be a rather difficult task for corporate managers of the health-care system. Their real challenge lies in achieving a successful alliance between providing optimal health care, patient satisfaction and the corporate goals of employee satisfaction in terms of pay and work ethics, in addition to cost containment and profits. The hospital has to become “at once a hotel, an industrial plant, a repair and rehabilitation shop, a haven of refuge and an educational institution” (Perkins 1998). It's far from the end of the road.

## Questions That This Paper Raises

1. Is the health-care system ailing? Is there an element of true satisfaction amongst the physicians when dealing with the corporate-run hospital or is there always an element of discontent?
2. Are physician payouts sufficient in the corporate setup or do the rising costs curtail adequate physician payout, whereas patient costs are still rising?
3. Is the rising influence of the medical-industrial nexus affecting the sacrosanct physician-patient relationship?
4. Can there, ever, be a solution to this timeless question, “*Do doctors necessarily, as often accused, compromise ethics in the pursuit of riches? Or is it just a few who give the profession a bad name?*”
5. Is the recent proliferation of corporate hospitals a healthy trend? With the tendency for costs to be high, does it make health care even more remote for the uninsured and middle-class patients?
6. Should the physician be made a part of every decision or cost-related amendment in the corporate setup?
7. Is there a need for a national policy on standards in private health care?
8. Should we as practitioners set up professional standards for our dealings with the corporate industry? Such parameters do exist with professional bodies abroad. Should similar policies on gifts and sponsorships be made standard operating procedure?
9. Is thinking of medicine as a *Patient Welfare-Centered Professional Enterprise* a practically workable solution?

## About the Authors

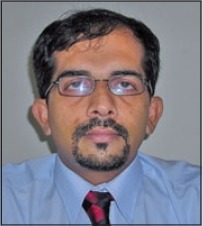



Dr. Murali Poduval MS, DNB is an alumnus of the Seth GS Medical College and the KEM hospitals, Mumbai, Dr. Murali Poduval is a senior lecturer in the department of orthopedics at these institutions. He is a keen follower of trends and influences in the health-care industry. He has special interest in writing and publishing and edits the IJCP's Asian Journal of Orthopedics and Rheumatology.

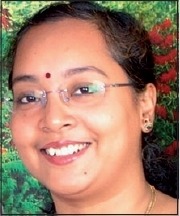



Dr. Jayita Poduval MS is an alumnus of the GS Medical College and the KEM hospitals, Mumbai. She is a practicing consultant in the field of ENT in the suburbs of Mumbai, with keen interest in nasal endoscopy. She spends her free time writing on both technical and public interest issues.
